# Construction and validation of a novel Ferroptosis-related gene signature predictive model in rectal Cancer

**DOI:** 10.1186/s12864-022-08996-6

**Published:** 2022-11-22

**Authors:** Wei-Kun Shi, Yu-Xin Liu, Xiao-Yuan Qiu, Jing-Ya Zhou, Jiao-Lin Zhou, Guo-Le Lin

**Affiliations:** 1 Department of General Surgery, Peking Union Medical College Hospital, Peking Union Medical College and Chinese Academy of Medical Sciences, Shuaifuyuan 1, Beijing, 100730 China; 2 Department of Medical Records, Peking Union Medical College Hospital, Chinese Academy of Medical Sciences and Peking Union Medical College, Beijing, China; 3 Collaborating Center for the WHO Family of International Classifications in China, Beijing, China

**Keywords:** Rectal cancer, Prognosis, Ferroptosis-related genes, Risk score

## Abstract

**Background:**

Rectal cancer (RC) is one of the most common malignant tumors. Ferroptosis is an iron-dependent form of cell death, which plays an important role in various cancers. However, the correlation between ferroptosis-related genes (FRGs) and prognosis in RC remains unclear.

**Methods:**

Gene expression data from The Cancer Genome Atlas Rectum adenocarcinoma (TCGA-READ) and GSE87211 were downloaded. Clustering and functional enrichment were evaluated. A FRGs risk score was established based on the univariate Cox analysis and the Least absolute shrinkage and selection operator (LASSO) analysis. K-M analysis and ROC analysis were conducted to determine prognostic values. qRT-PCR was performed to validate levels of mRNA expression. Multivariate Cox analysis was used to build a prognostic prediction model based on the risk score.

**Results:**

Based on FRGs, RC patients were grouped into two clusters. In the functional enrichment of differentially expressed genes between the two clusters, immune-related pathways dominated. A novel FRGs signature with 14 genes related to the overall survival (OS) of RC was established. qRT-PCR of the 14 genes identified TP63, ISCU, PLIN4, MAP3K5, OXSR, FANCD2 and ATM were overexpressed in RC tissue; HSPB1, MAPK1, ABCC1, PANX1, MAPK9 and ATG7 were underexpressed; TUBE1 had no difference. The high-risk group had a significantly lower OS than the low-risk group (*P <* 0.001), and ROC curve analysis confirmed the signature’s predictive capacity. Multivariate analysis demonstrated that the risk score and age were independent prognostic factors.

**Conclusion:**

A novel FRGs model can be used to predict the prognosis in RC, as well as to guide individual treatment.

**Supplementary Information:**

The online version contains supplementary material available at 10.1186/s12864-022-08996-6.

## Introduction

Colorectal cancer (CRC) is the third most prevalent cancer worldwide, and rectal cancer (RC) accounts for approximately 40% of all CRCs [1]. RC is the eighth leading cause of cancer-related mortality globally, with about 339,022 deaths in 2020 [2].

Due to the popularization of digestive endoscopy and the development of MRI, endoscopic ultrasound and other technologies, early diagnosis of RC has been greatly improved [3, 4], and the method of treatment has also been developed from a single TME (total mesorectal excision) radical operation to a multidisciplinary combined treatment modality including neoadjuvant chemoradiotherapy (nCRT) along with organ-sparing surgery [5]. However, the prognosis of RC remains unsatisfactory. Despite nCRT combined with radical surgery and adjuvant chemotherapy, the long-term distant metastasis rate after surgery can still reach 30% [6], and the 10-year survival rate may be below 50% [7].

Some molecular biological diagnostic techniques have been applied to diagnose, treat and predict the prognosis of RC, such as monitoring circulating tumor DNA (ctDNA) expression levels to predict the prognosis [8], detecting UGT1A1 gene polymorphisms to predict the chemotherapy sensitivity [9], detecting microsatellite instability to predict the efficacy of immune checkpoint inhibitors (ICI) [10]. With the ultimate goal of improving the patient’s prognosis, these technologies can make the treatment of RC more accurate. Hence, an in-depth discussion of the diagnosis and treatment of RC at the molecular level is the current research hotspot and the direction of future development. We need to further explore molecular targets for the diagnosis and treatment of RC. In addition, we need to provide novel methods for the diagnosis, treatment, and prognostic prediction of RC patients through various mechanisms.

Ferroptosis is an iron-dependent programmed cell death pattern characterized by the accumulation of lipid peroxides, proposed by Stockwell et al. in 2012 [11]. The core molecular mechanism of ferroptosis includes regulating the balance between oxidative damage and antioxidant defense, which requires the precise regulation of ferroptosis-related genes (FRGs) and their expression products, and this balancing process also greatly affects the occurrence and development of tumor cells [12]. Numerous studies have explored the relationship between ferroptosis and malignant tumors at the genetic level, such as trying to treat renal clear cell carcinoma by glutathione peroxidase 4 (GPX4)-dependent ferroptosis [13], inhibiting ALOX5 may promote ferroptosis in pancreatic ductal adenocarcinoma [14], SLC7A11-mediated cystine uptake inhibits ferroptosis in breast cancer cells [15] etc. Based on a large number of studies, some researchers have summarized the related literature on ferroptosis, and classified the genes involved into a database [16]. This may provide better support for our research on the mechanism of ferroptosis.

The clinical application of ferroptosis focuses on targeting the expression of some FRGs to specifically induce ferroptosis in tumor cells, thus obtaining anti-cancer effects [17, 18]. In order to obtain detailed information about a target, it is usually necessary to perform bioinformatics analysis to determine whether tumor tissue differs from normal or paracancerous tissues. In CRC, there are some studies discussing the role of FRGs, for example, activation of ATF3 may promote ferroptosis by inhibiting the Xc^−^ system [19]; CDKN2A sensitizes cancer cells to ferroptosis by downregulating SLC7A11 [20]. Some studies also use a set of FRGs to develop a risk model for predicting the prognosis of CRC [21, 22]. However, such studies on ferroptosis and RC alone are lacking.

It is necessary for us to analyze FRGs in RC, find ferroptosis-related markers, and evaluate their prognostic value, so as to provide reference for further basic research and clinical translation.

## Materials and methods

### Data source

The Cancer Genome Atlas (TCGA) (https://portal.gdc.cancer.gov/) contains 167 rectum adenocarcinoma (READ) tumor samples and GSE87211 acquired from Gene Expression Omnibus (GEO) (https://www.ncbi.nlm.nih.gov/geo/) which include 196 RC patients were used as training set and external validation sets, respectively. We obtained human FRGs from the FerrDB database (http://www.datjar.com:40013/bt2104/). The Human Protein Atlas (HPA) (https://www.proteinatlas.org/about/download) was used to verify the immunohistochemical (IHC) staining of genes.

### Clustering and identification of differentially expressed genes (DEGs)

The ConsensusClusterPlus R package [23] was used to perform unsupervised clustering and divide the RC into two clusters according to the FRGs. This method is based on an algorithm called consensus clustering, which can provide quantitative evidence for determining the number of potential clusters within the RNA-seq data. The t-distributed stochastic neighbor embedding (t-SNE) analysis was applied based on FRGs to visualize the data in two dimensions with the Rtsne and ggplot2 packages [24]. Then, the limma R package [25] was used to analyze the ferroptosis-related DEGs (FRDEGs) between these two groups. FRDEGs were identified based on *P* value < 0.05 and the absolute value of Fold Change > 1 (|Log2fold change| > 1). Volcano plots and a Venn diagram were drawn with the ggplot2 R package. The heat map was generated by the pheatmap R package.

### Gene ontology (GO) and Kyoto encyclopedia of genes and genomes (KEGG) analysis

GO analysis, including biological process (BP), cellular composition (CC) and molecular function (MF), was performed for FRDEGs characteristics using the clusterProfiler R package [26]. We also used this package to analyze the functional enrichment of the FRDEGs in KEGG pathways [27].

### Establishment and validation of the risk score based on the FRGs

The univariate Cox regression analysis was performed to determine the FRGs with the prognostic value, and the cutoff *p*-value was set at 0.05. Then, least absolute shrinkage and selection operator (LASSO) regression was performed to further screen relevant key genes. Ten-fold cross-validation was used to select the most suitable model parameters λ. Next, we used these key genes to calculate a risk score, which was obtained using the formula: risk score = (βA × Gene A expression) + (βB × Gene B expression) … + (βN × Gene N expression). According to the median risk score, RC patients were divided into two groups: the low-risk group and the high-risk group. The survival differences between the low- and high-risk groups were compared through Kaplan-Meier (K-M) analysis. In addition, we also performed receiver operating characteristic (ROC) analysis to access the survival predictive ability of this risk score. The GEO dataset was used as a validation group to verify the above outcomes.

### Quantitative real-time PCR (qRT-PCR)

qRT-PCR was performed on 10 pairs of RC tissues and adjacent normal tissues to validate the mRNA expression levels of the 14 signature genes. Consent forms were obtained from each patient for the collection and analysis of tissue samples. The study was approved by the Peking Union Medical College Hospital ethics review boards. We immediately froze and stored the tissues in liquid nitrogen after extracting them. Total RNA was extracted from the sample tissues via Trizol lysate (Thermo Fisher Scientific), followed by reverse transcription to cDNA. qRT-PCR was carried out using the CFX96 system (BIO-RAD CFX96; BIO-RAD Laboratories, Inc., Hercules, CA, USA). GAPDH served as an internal control. Relative expression levels were quantified by the Ct (2^−ΔΔCt^) method, and the mean value was used as the final experimental result for replicated wells. All procedures were carried out in accordance with the manufacturer’s instructions.

### Gene set enrichment analysis (GSEA)

To explore the different KEGG pathways between high- and low-risk groups, GSEA was conducted with the Molecular Signatures Database (MSigDB). Pathways with an adjusted *P* < 0.05 were identified as significant enrichment pathways in various risk groups.

Evaluation of immune microenvironment characteristics, immune checkpoint related characteristics and chemotherapy drugs’ sensitivity.

xCELL [28] algorithm was used to estimate the abundance scores of 64 immune cells per sample. Tumor mutation burden (TMB) was calculated according to the mutation information from TCGA READ cohort. The TMB estimate for each sample is equal to the total mutation frequency/38, since 38 Mb is routinely taken based on the length of the human exon. To predict the effect of immune checkpoint blockade therapy, the expression of 34 potential immune checkpoint genes was analyzed. Furthermore, the Tumor Immune Dysfunction and Exclusion (TIDE) (http://tide.dfci.harvard.edu/) algorithm was used to estimate the ICIs response of RC patients from TCGA cohort [29]. The pRRophetic R package [30] was used to evaluate the sensitivity of chemotherapy drugs. Compare the above characteristics of high- and low-risk groups for diagrams.

### Establishment of the prognostic model

Univariate and multivariate Cox regression analyses were performed to establish the prognostic model based on the risk score and other clinicalpathological characteristics (age and TNM stage) of the patients. We constructed a nomogram to predict the overall survival (OS) of RC patients in 1-, 3- and 5-year according to the prognostic model. Then, we used the median total points for each patient based on this nomogram, to divide patients into high-risk and low-risk groups. The K-M curve was plotted and ROC analysis was carried out at 1-, 3- and 5-year of survival. Next, calibration curves were used to estimate whether the predicted survival results of the nomogram (1-, 3- and 5-year survival) were close to the actual results.

### Statistical analysis

All analyses were performed using R version 4.1.3 (https://www.r-project.org/) and its appropriate packages. Univariate, LASSO and multivariate Cox regression analyses were conducted to build models. Log-rank test was used in the K-M analysis. Two groups were adjusted by the Wilcoxon test and the *p*-value was corrected by Benjamini and Hochberg (BH) test. Two-sided *P <* 0.05 indicated statistical significance.

## Results

FRGs were clustered in patients with RC, followed by the identification of DEGs and functional enrichment analysis.

**Fig. 1:**
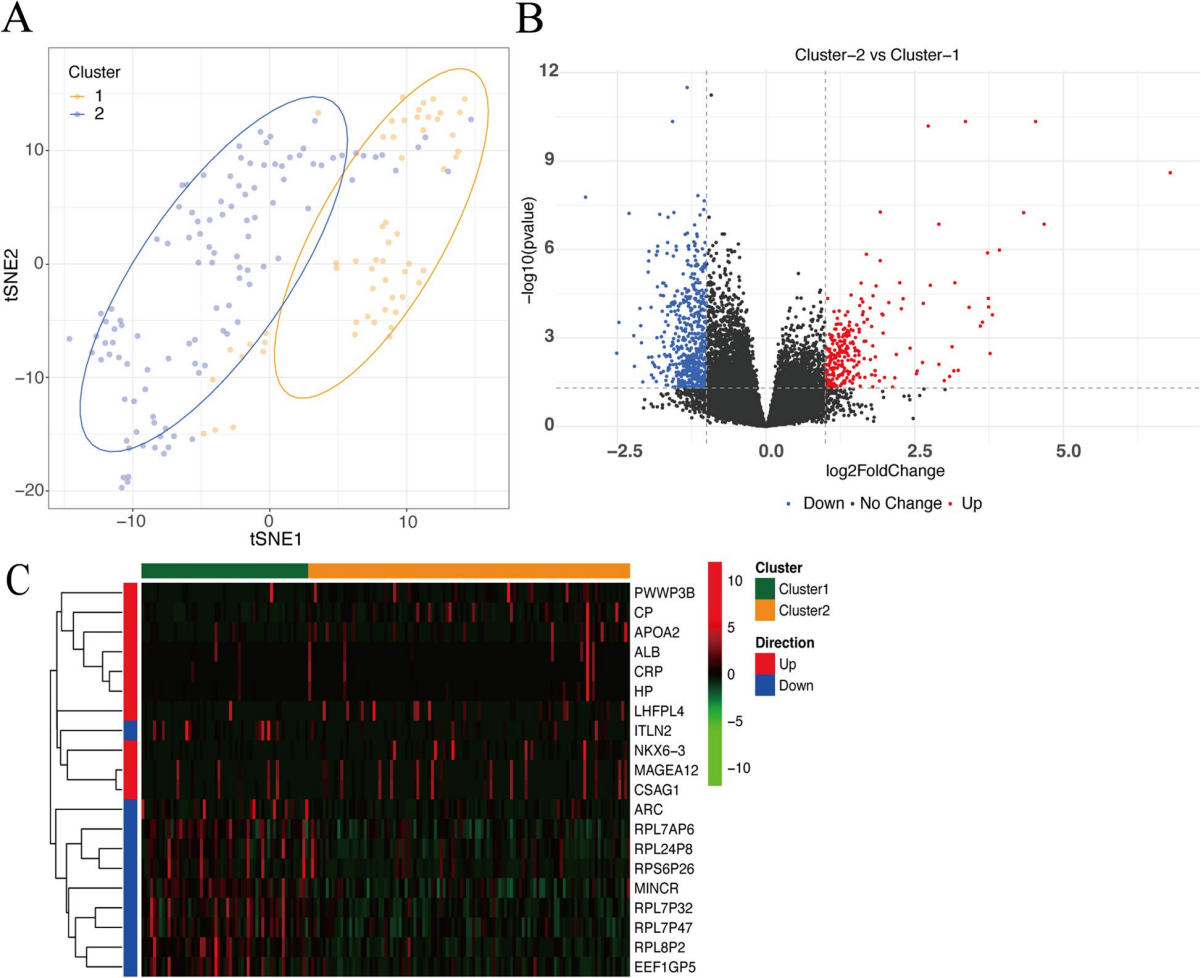
**a** Clustering tumor samples into two classes based on FRGs in RC. **b** The volcano plot for the differential expression analysis according to different clusters. **c** The heatmap of the top 10 up-regulated and down-regulated FRGs

Cluster analysis of TCGA READ gene expression data using FRGs can divide RC patients into two clusters (Cluster 1 and Cluster 2). Visualization of the two groups after dimensionality reduction was shown in Fig. 1(a). We conducted the subsequent analysis based on two clusters, and also provided consensus matrix heatmaps of 3 to 9 clusters for readers’ reference (Supplementary Fig. 1). We obtained 822 DEGs, 263 genes were up-regulated and 559 were down-regulated (Fig. 1(b)). The heatmap of the top 10 up-regulated and top 10 down-regulated genes was shown in Fig. 1(c).

**Fig. 2:**
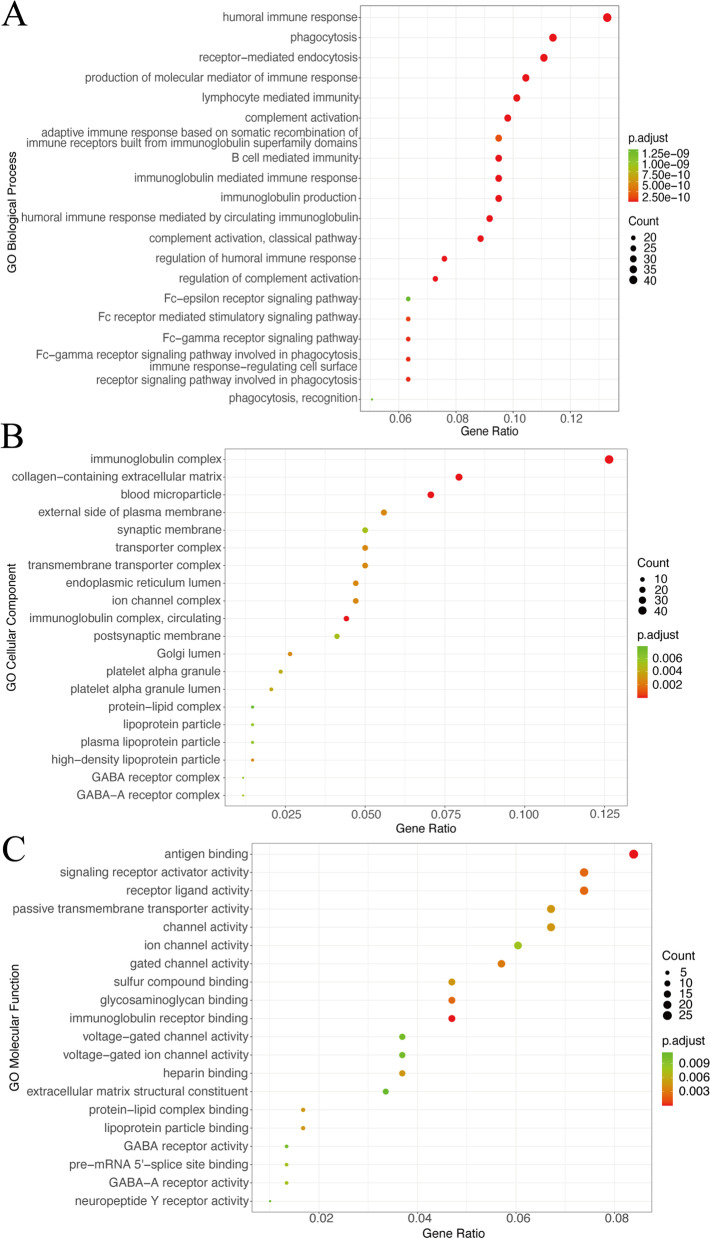
**a**-**c** BP, CC and MF of GO analysis

**Fig. 3:**
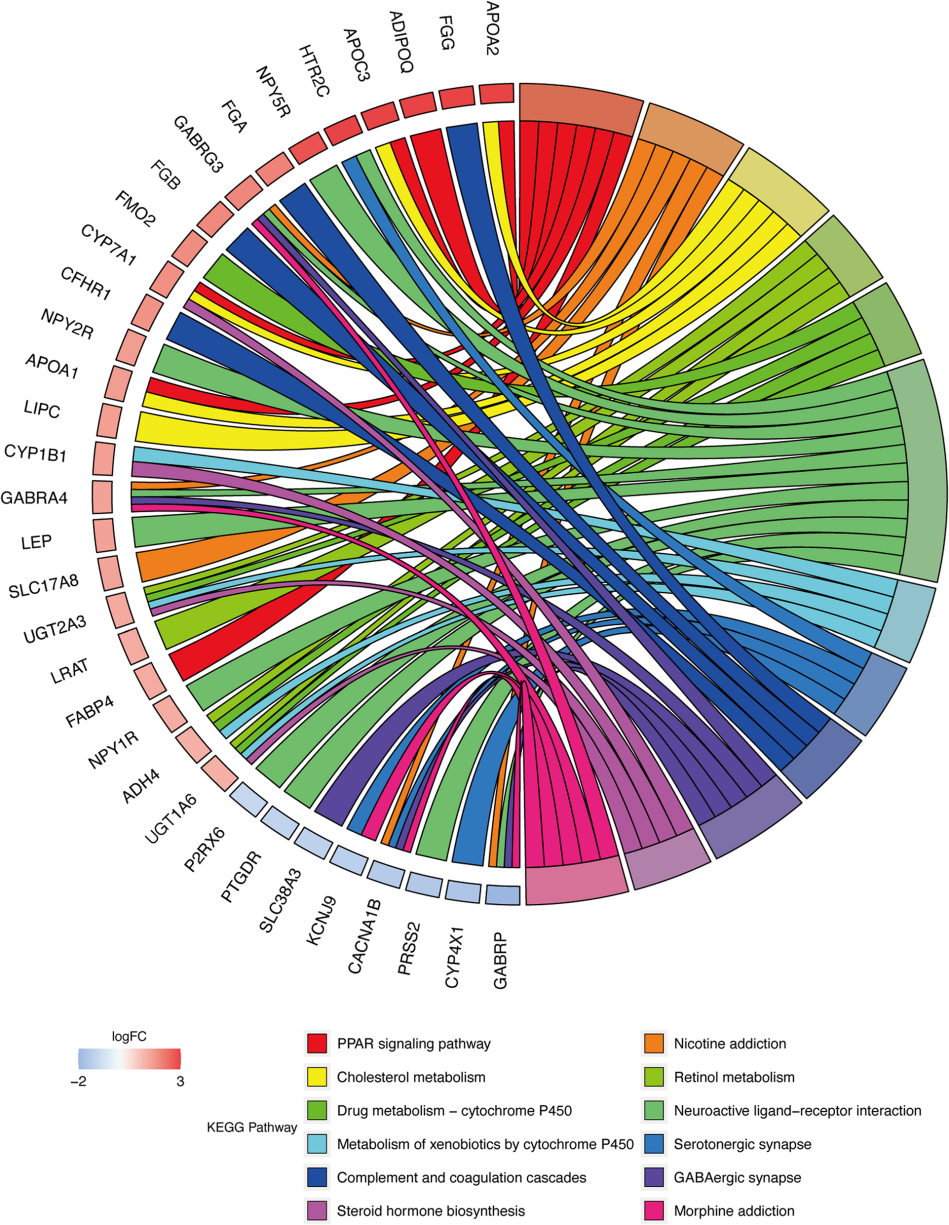
(A) KEGG enrichment analysis

The results of the GO enrichment analysis were presented in Fig. 2(a-c), which showed the enrichment of the 20 most significant pathways (ranked according to the adjusted *p*-value) in BP, CC and MF respectively. Differential genes were enriched in immune-related pathways in each facet, and were additionally associated with phagocytosis, transmembrane transport, GABA receptors, ion channels etc. The result of the KEGG enrichment analysis was shown in Fig. 3. As with the GO enrichment analysis, differential gene expression was associated with immune pathways using KEGG enrichment analysis. This analysis also found associations with PPAR, retinol metabolism, P450 enzymes and drug metabolism, and the nervous system.

### Establishment of a risk score

**Fig. 4:**
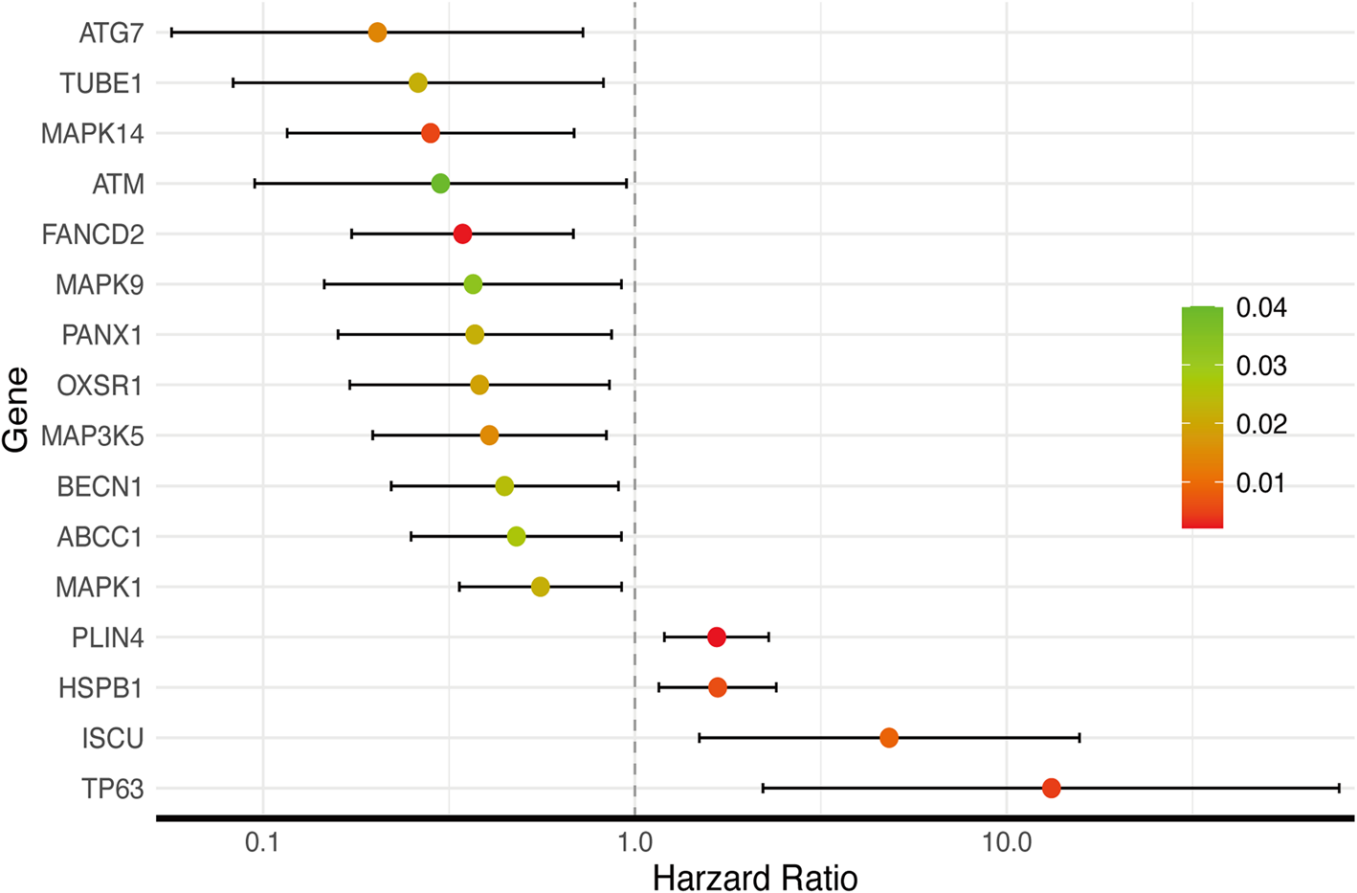
The univariate Cox regression analysis of the training group show 16 FRGs were correlated with OS

**Fig. 5:**
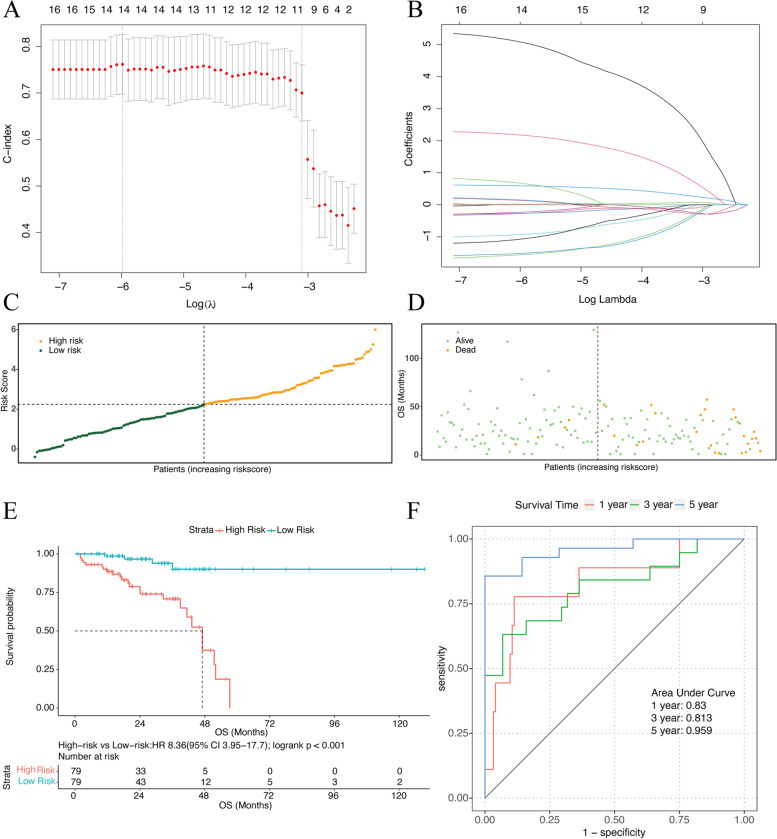
**a** Screening for the best LASSO model parameter λ. **b** Variable number change alongside different λ. **c** Distribution of risk scores. **d** Survival status of patients with different risk scores. **e** K-M plot of the training dataset. **f** The ROC curves of 1-, 3-, and 5-year OS

Using univariate Cox regression analysis with FRGs in the training group, 16 genes were correlated with the OS of RC patients (*P <* 0.05) (Fig. 4). To prevent model overfitting, 14 genes (TP63, ISCU, HSPB1, PLIN4, MAPK1, ABCC1, MAP3K5, OXSR1, PANX1, MAPK9, FANCD2, ATM, TUBE1 and ATG7) were selected by LASSO regression (Figs. 5(a) and 5(b)). When the minimum λ value was 0.002521672, the following risk score formula was obtained: Riskscore = (5.076640326) * TP63 + (2.175090008) * ISCU + (0.018180284) * HSPB1 + (0.593316672) * PLIN4 + (0.155545632) * MAPK1 + (0.136338804) * ABCC1 – (0.271718945) * MAP3K5 + (0.670705631) * OXSR1 – (0.266461901) * PANX1 – (0.928347939) * MAPK9 – (0.230410588) * FANCD2 – (1.069047524) * ATM – (1.559344596) * TUBE1 – (1.51520076) * ATG7. Based on the median of the risk score, we separated the cases into two groups: high-risk and low-risk groups (Fig. 5(c)). The risk distribution plot revealed that the high-risk group had significantly higher mortality and shorter OS than the low-risk group (Fig. 5(d)). The K-M curve indicated that the high-risk group had a significantly worse OS (*P <* 0.001) with a hazard ratio (HR) of 8.36 (95%CI 3.95–17.7) (Fig. 5(e)). The area under curves (AUCs) of 1-, 3-, and 5-year OS were 0.83, 0.813 and 0.959 according to ROC curves (Fig. 5(f)).

### Validation of the risk score using the GEO cohort

**Fig. 6:**
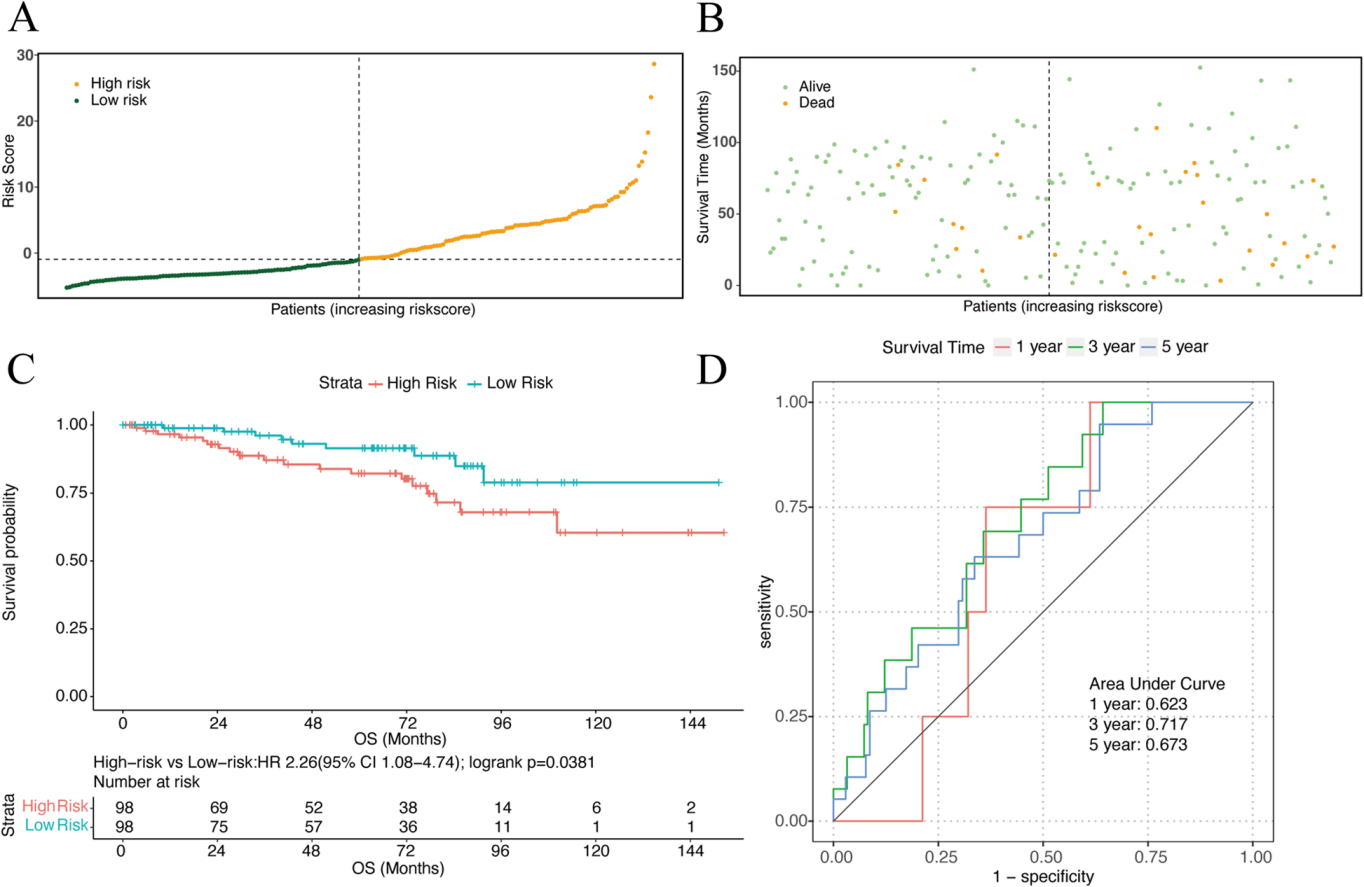
**a** Distribution of risk scores in validation dataset. **b** Survival status of patients with different risk scores. **c** K-M plot of the validation dataset. **d** The ROC curves of 1-, 3-, and 5-year OS in validation dataset

We then evaluated the prognostic efficiency of the risk model by analyzing the data of RC from the GEO dataset (GSE87211). The distribution of risk scores and survival status in the dataset is presented in Fig. 6(a) and 6(b). Likewise, as the risk score increased, the number of deaths also increased. Similar to the results from the TCGA cohort, the high-risk group had a significantly poorer OS in the GEO dataset (*P* = 0.0381) with a HR of 2.26 (95%CI 1.08–4.74) (Fig. 6(c)). The AUCs for 1-, 3- and 5-year OS were 0.623, 0.717 and 0.673, respectively, in the GEO dataset (Fig. 6(d)).

### IHC differences using the HPA database

**Fig. 7:**
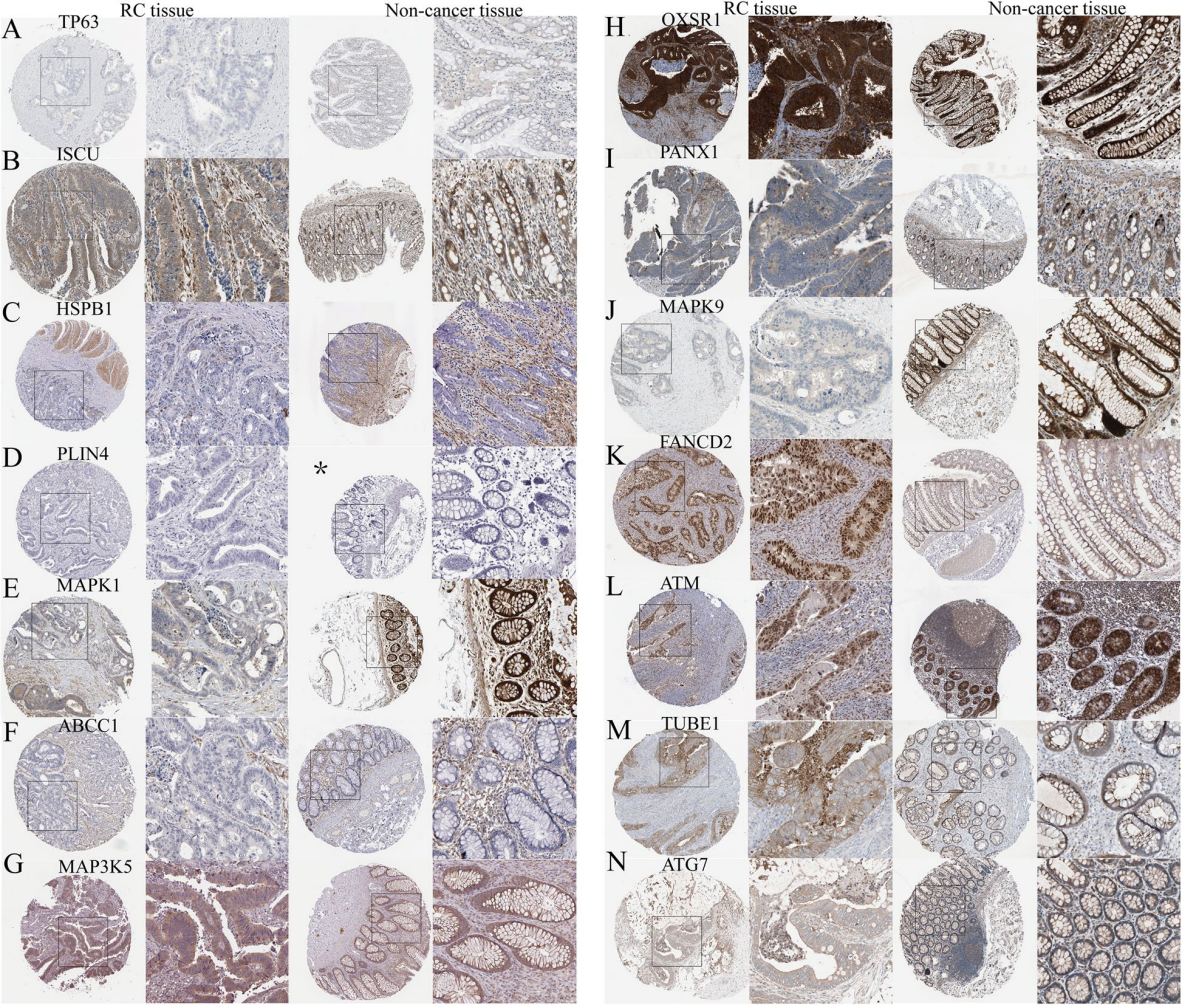
IHC differences between RC tissue and normal tissue using the HPA database. **a**-**n** IHC of the 14 genes in the risk score. *using colon tissue instead due to lack of rectal tissue

To further confirm the importance of the 14 FRGs in the risk score, HPA database was used to compare their protein expression in non-cancerous and RC tissues. According to Fig. 7, only ATM was highly expressed in RC tissue as compared to normal rectal tissue. In contrast, HSPB1, MAPK1, ABCC1, PANX1, MAPK9 and ATG7 were downregulated in RC. The protein levels of TP63, ISCU, PLIN4, MAP3K5, OXSR1, FANCD2 and TUBE1 had no significant changes between RC and normal rectal tissue.

### qRT-PCR

**Fig. 8:**
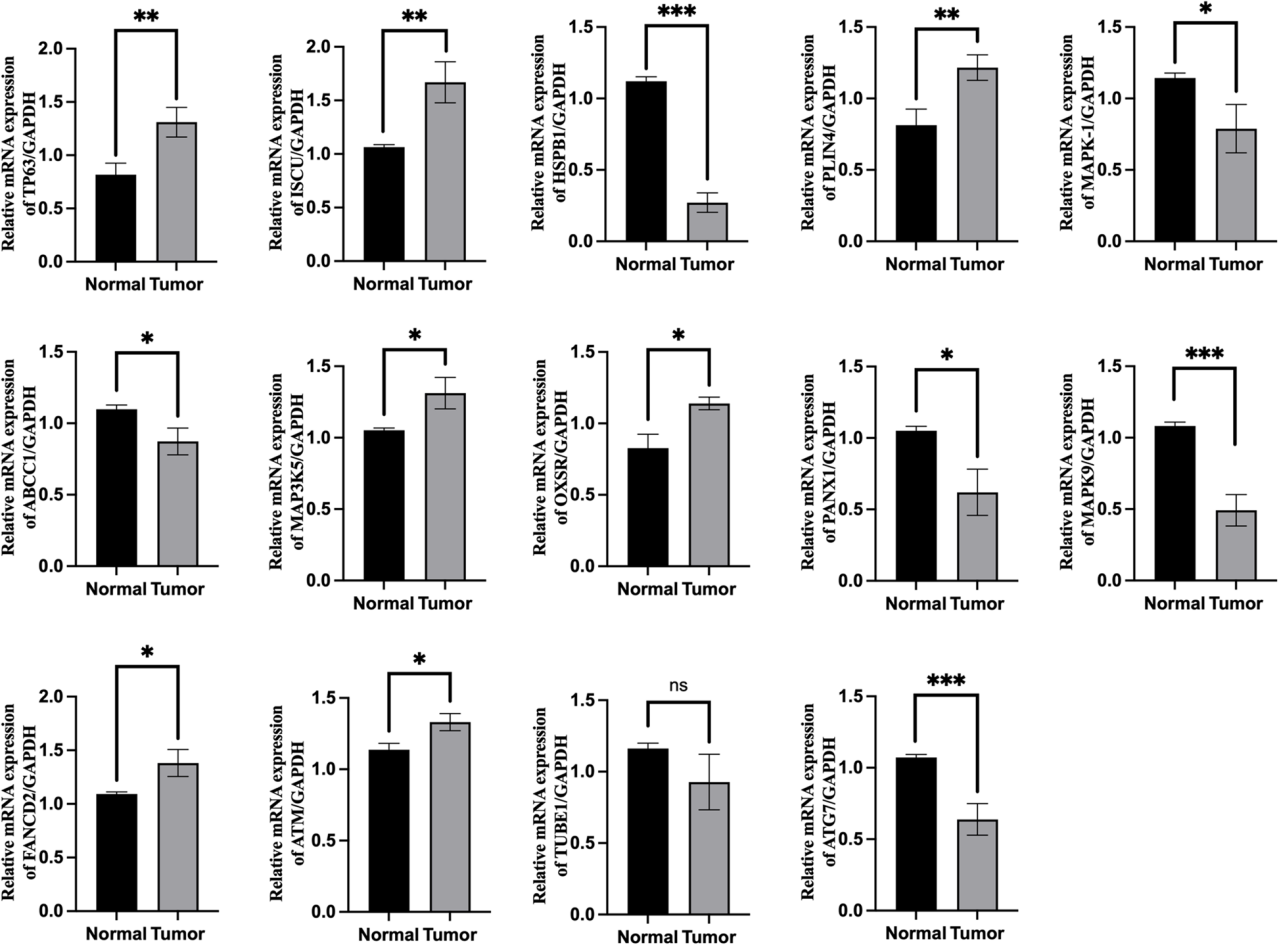
The relative expression levels of the 14 genes in normal and RC tissues by qRT-PCR. (*P < 0.05; **P < 0.01, ***P < 0.001, *ns* not significant)

In RC tissue, the expression of TP63, ISCU, PLIN4, MAP3K5, OXSR, FANCD2 and ATM was significantly higher than that of normal tissue. In contrast, the expression of HSPB1, MAPK1, ABCC1, PANX1, MAPK9 and ATG7 was significantly lower than that of normal tissue. There was no significant difference in the expression of TUBE1. (Fig. 8).

### GSEA

**Fig. 9:**
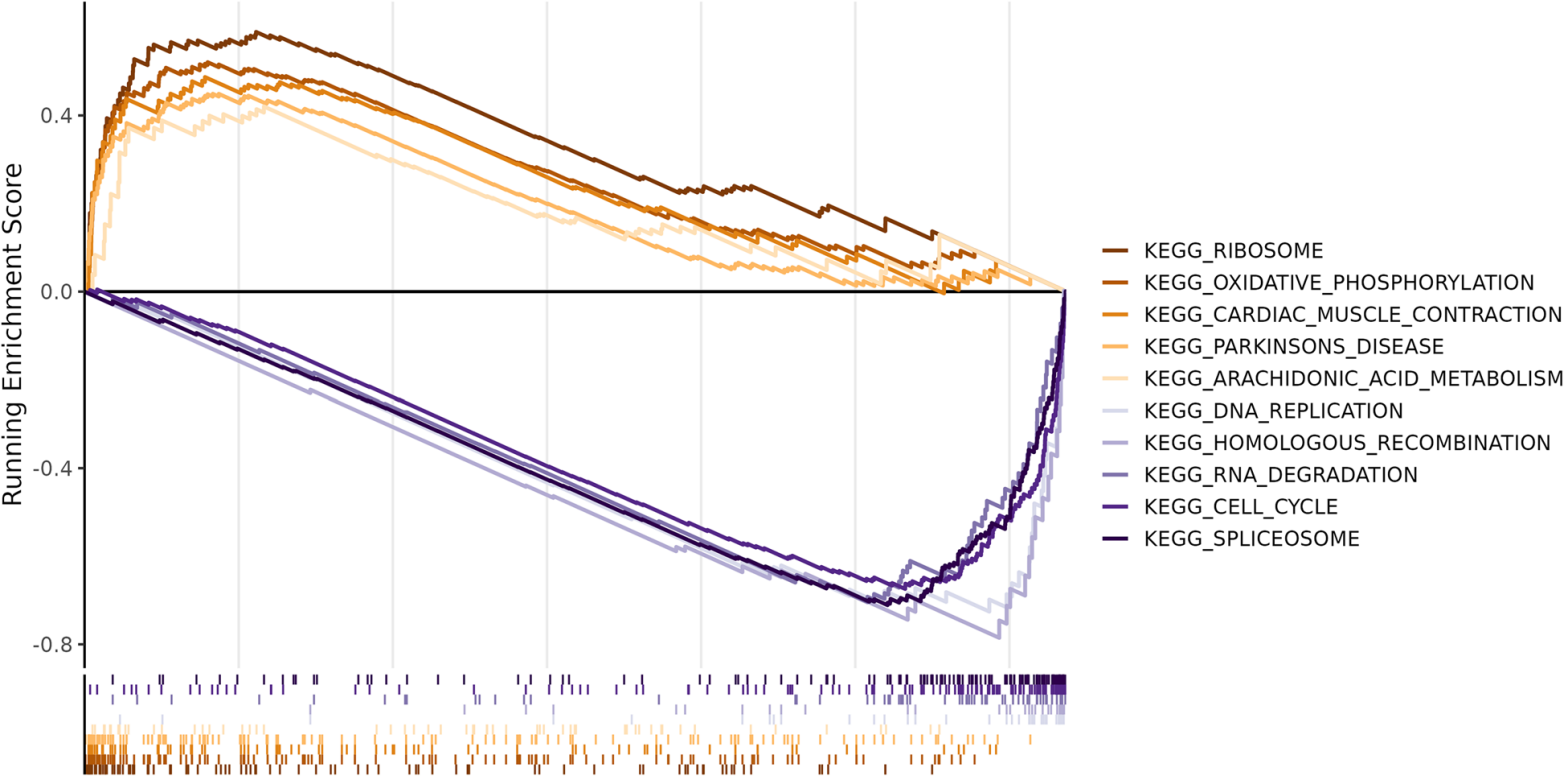
GSEA of different KEGG pathways between high- and low-risk groups

The pathways of ribosomes, oxidative phosphorylation, and arachidonic acid metabolism were mainly enriched in high-risk groups. In contrast, the pathways of DNA replication, homologous recombination, RNA degradation, cell cycle and spliceosome were mainly enriched in the low-risk group (Fig. 9).

### Prognostic FRDEGs had minor effects on the immune microenvironment of RC

**Fig. 10:**
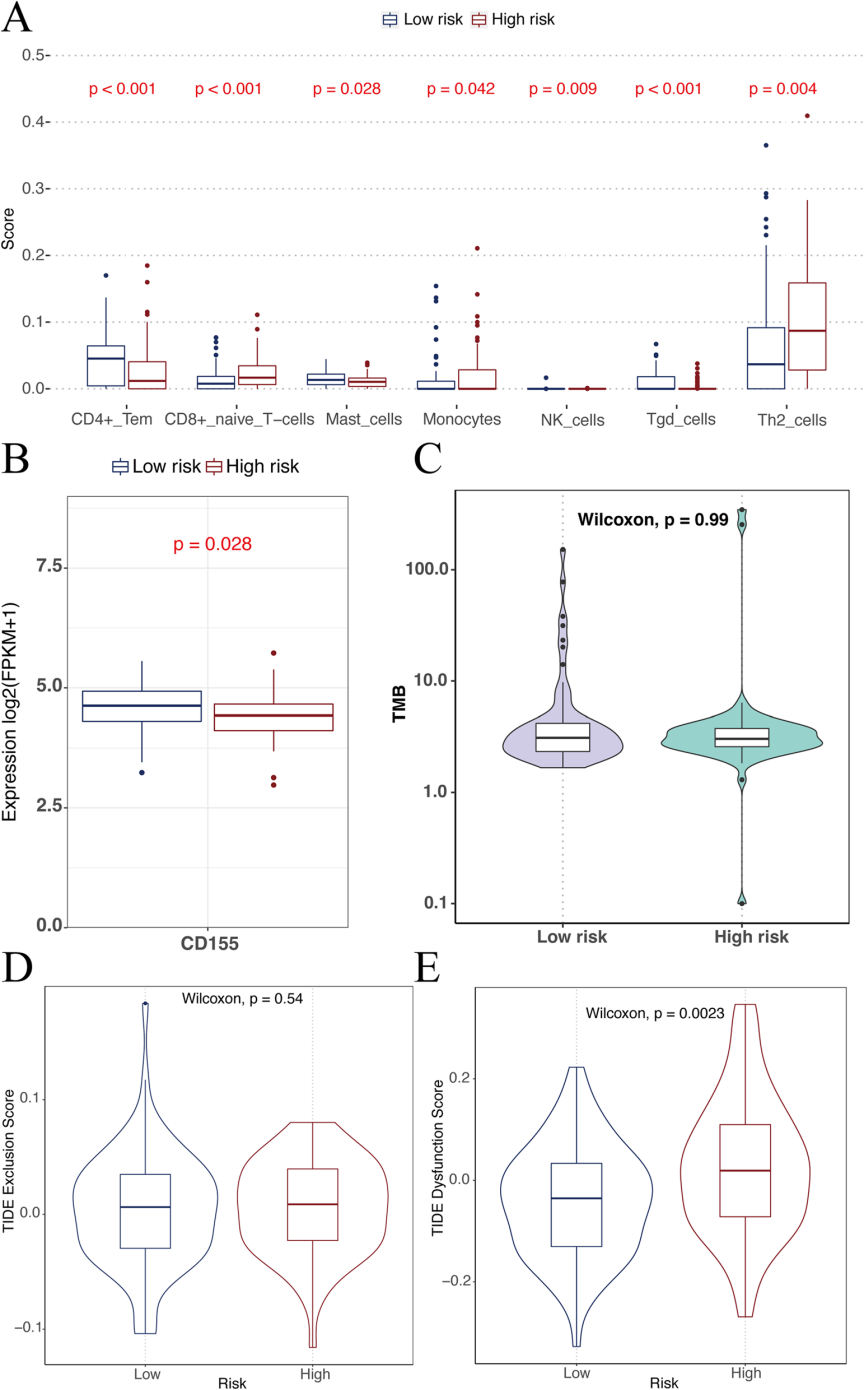
**a** Difference of immune cells’ scores between high- and low-risk groups. **b** Differential analysis of immune checkpoint expression between two groups. **c** Difference of TMB between two groups. **d**-**e** Difference of TIDE scores between two groups

We compared immune infiltration by xCELL between patients in the low- and high-risk groups and observed that the infiltration levels of CD4^+^ Tem, CD8^+^ naive T-cells, mast cells, monocytes, NK cells, Tgd cells, Th2 cells were significantly different (Fig. 10(a)). There was no statistical difference in TMB between the high- and low-risk groups (Fig. 10(b)). The TIDE analysis data showed that the high-risk group had a statistically larger dysfunction score than the low-risk score, while the exclusion score had no difference (Fig. 10(c) and (d)). In the evaluation of 34 immune checkpoint gene expressions, only CD155 was significantly different (Fig. 10(e)).

### Analysis of the relationship between the risk model and chemotherapy

**Fig. 11:**
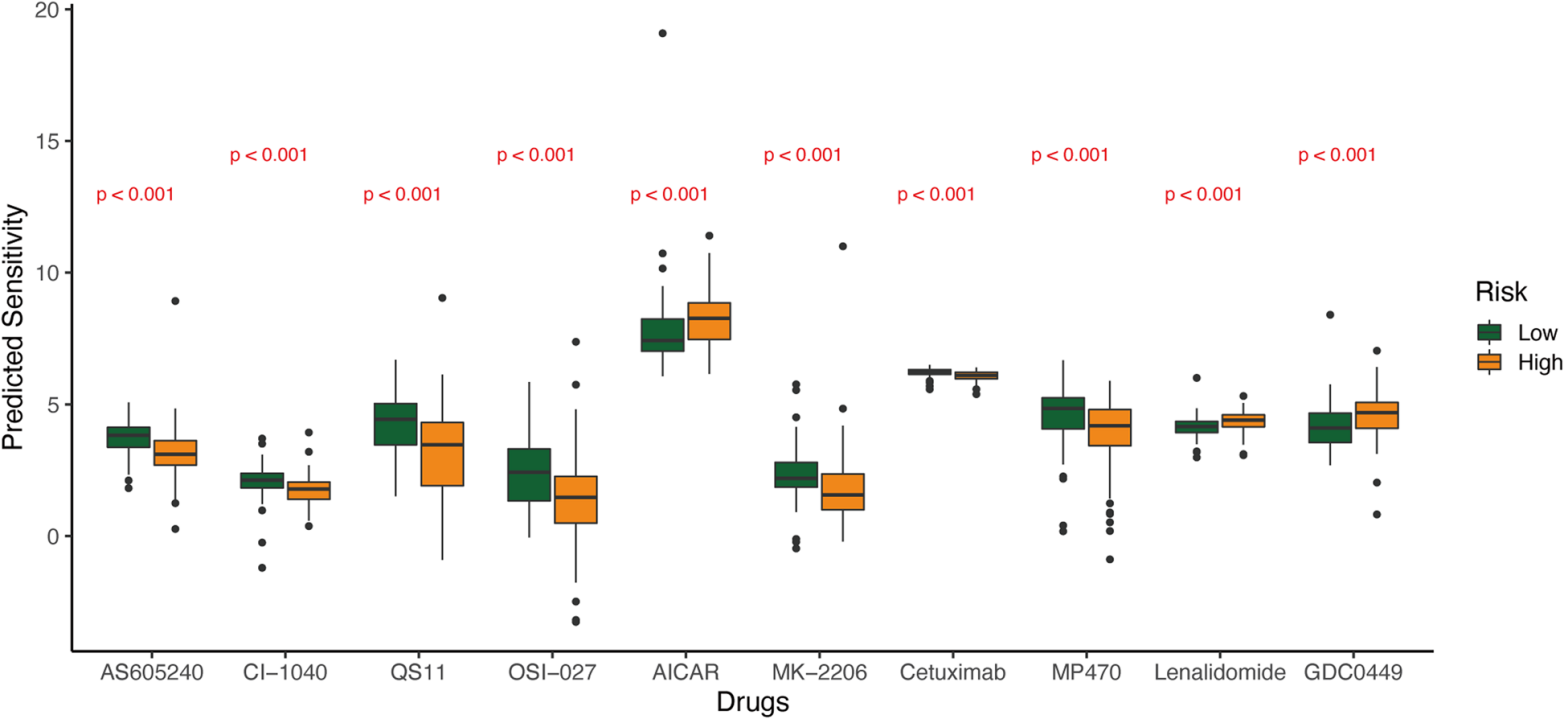
Differential analysis of chemotherapeutic drugs’ response between high and low risk groups

The results showed that there were some chemotherapeutic drugs with significantly different IC50s between the high- and low-risk groups, among which the more sensitive drugs in the high-risk group were: AS605240, CI-1040, QS11, OSI-027, MK-2206, Cetuximab and MP470 (Fig. 11).

### Construction of the nomogram

**Fig. 12:**
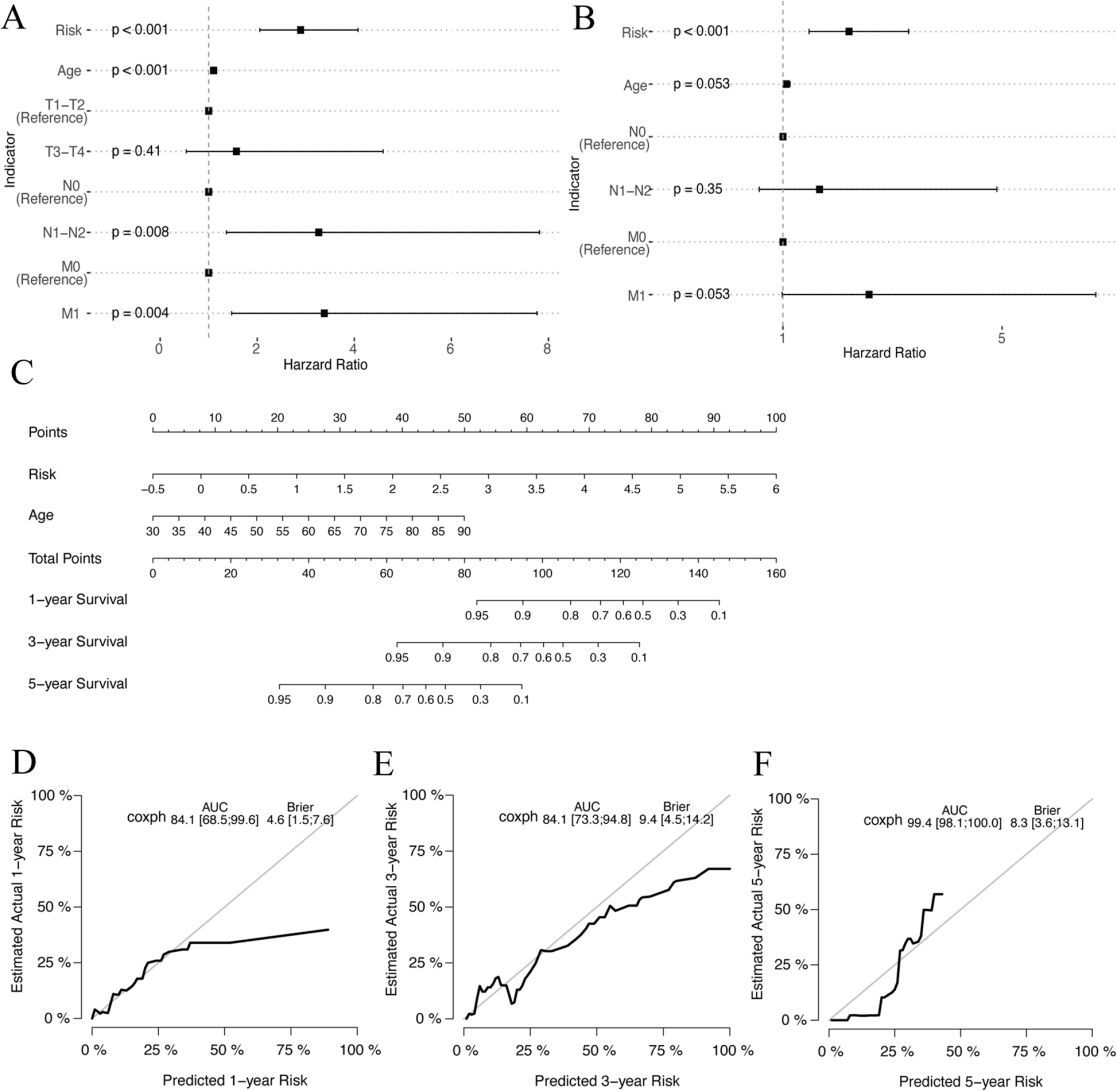
**a**-**b** Univariate and multivariate Cox analysis of the risk score and clinical traits in training dataset. **c** The prognostic nomogram predicting 1, 3 and 5-year OS. **d**-**f** Calibration curves (grey line) of 1, 3 and 5-year OS in risk model

Based on the univariate and multivariate Cox regression analysis of the risk score and clinical traits (age and TNM stage) (Fig. 12(a) and (b)) in the training cohort, we built a prognostic nomogram to predict 1, 3 and 5-year OS probabilities of RC patients (Fig. 12(c)). The calibration curves demonstrated that the model’s predictions of 1, 3 and 5-year OS probabilities were favorably consistent with the ideal predictions (Fig. 12(d-f)).

## Discussion

RC is a dangerous malignant tumor that threatens human health. At present, the efficacy of comprehensive treatment needs to be improved. Many molecular mechanisms are still being explored, and many reasons for the occurrence and development of RC are still unclear. Ferroptosis is an iron-dependent form of cell death. In the field of malignant tumors, people hope to inhibit tumor development by promoting ferroptosis in tumor tissue, and providing new targets and a pharmacological basis for tumor therapy.

In this study, the risk score of FRGs in RC was established by analyzing the transcriptome, clinical information, and survival information of TCGA READ. A risk prediction model was built on the basis of the risk score, combined with multivariate Cox analysis. The model can effectively predict the 1-, 3- and 5-year OS of RC, providing a potentially useful tool for clinical practice and further mechanism research.

First, we performed a cluster analysis on TCGA READ samples from the perspective of FRGs. The clustering results were divided into two categories, meaning that the RCs were divided into two different types of ferroptosis gene expression patterns. We use GO enrichment analysis and find that these FRDEGs are mainly enriched in immunity in BP, CC and MF. This suggests that the differences in gene expression of ferroptosis in RC patients are closely related to immune responses. Many studies have shown that ferroptosis is closely associated with the tumor immune microenvironment [31]. The study by Fushun et al. found that BEBT-908-induced ferroptosis results in upregulation of MHC I and activation of endogenous IFNγ signaling in tumor cells, thereby improving cancer immunotherapy [32]. There will be further studies on ferroptosis and cancers, including RC, from the perspective of immunotherapy, in an effort to benefit patients.

To further investigate FRGs affecting RC, univariate Cox regression was applied to screen genes significantly associated with OS. Then, 14 genes were identified using LASSO regression to establish a risk score. We divided patients into the high- and low-risk groups by the risk score, and survival analysis indicated that the prognosis of the high-risk group was significantly worse. ROC analysis showed that this risk score was very accurate in predicting OS at 1, 3, and 5 years in the TCGA READ cohort. Therefore, we selected another RC cohort from GEO for external validation. The results showed that there was still a significant difference in OS between the two groups. The accuracy of OS prediction, though decreased, was still above 0.6, indicating that the risk score has reasonable extrapolation ability. We briefly checked the expression of the 14 genes encoded proteins through the HPA database, and found that only ATM was significantly highly expressed in RC tissue. Then, we performed qRT-PCR analysis of these 14 genes with surgical specimens from our research center, and the results showed that TP63, ISCU, PLIN4, MAP3K5, OXSR, FANCD2 and ATM were significantly highly expressed in tumor tissue. The reasons for the different results of IHC and qRT-PCR include: the sensitivity of IHC is not high, resulting in the failure to identify proteins with significant differences; there are some additional intervening factors in the process from transcription to translation, which need to be further explored.

We briefly introduce these 14 genes one by one. TP63 encodes a member of the p53 transcription factor family, and studies have shown that up-regulation of TP63 may further activate the glutathione metabolic pathways, including GPX4, thus reducing cellular exposure to oxidative stress, which supports the survival of tumor cells [33, 34]. ISCU encodes a component of the iron-sulfur (Fe-S) cluster scaffold. As an important redox center, iron-sulfur clusters have participated in numerous physiological functions, particularly in the metabolism [35]. HSPB1 encodes a member of the small heat shock protein (HSP27), and its expression has been demonstrated to inhibit the ferroptosis process of tumor cells, thereby promoting tumor growth [36]. PLIN4 is linked to the formation of lipid droplets which store intracellular free fatty acids and inhibit lipotoxic cell death. Studies have shown that PLIN4 upregulation can increase the viability and drug resistance of tumor cells [37], and this process is likely to be closely related to the inhibition of ferroptosis. MAPK1, MAP3K5 and MAPK9 belong to MAPK-signaling systems which play major roles in tumors. They provide a connection between transmembrane signaling and changes in transcription in response to different environmental signals such as cytokines, growth factors, oxidative stress and inflammation [38]. Ferroptosis of certain sensitive cell lines may be blocked by MAPK inhibition [18]. Tumors with sustained MAPK activation are capable of responding to in vivo cystine depletion by inducing ferroptosis [39]. ABCC1 encodes multidrug resistance-associated protein 1 (MRP1) is a member of the superfamily of ATP-binding cassette (ABC) transporters. Studies have shown that cells overexpressing MRP1 can disrupt GSH homeostasis and hinder GPX4 activity, thereby making cells more susceptible to ferroptosis [40]. OXSR1 encodes a serine–threonine protein kinase, and studies have shown that high OSR1 expression causes an increase in deaths specifically attributed to breast cancer and is related to an increase in lymph node metastasis [41]. However, the specific mechanism remains to be explored. PANX1 encodes an ATP-releasing pathway family protein and PANX1 deletion protects against renal ischemia/reperfusion injury by attenuating the MAPK/ERK activation in a ferroptotic pathway [42]. FANCD2 encodes a nuclear protein involved in DNA damage repair. Studies have shown that it can protect against ferroptosis-mediated injury in bone marrow stromal cells [43]. In addition, it is significantly overexpressed in lung cancer tissue and is associated with the prognosis of lung cancer [44]. ATM encodes an important cell cycle checkpoint kinase. ATM inhibition rescued ferroptosis by increasing the expression of iron regulators involved in the storage and export of iron [45]. TUBE1 encodes a tubulin superfamily member that was included in a ferroptosis prognostic model of skin cutaneous melanoma [46]. ATG7 encodes an activation enzyme that is essential for autophagy and cytoplasmic to vacuole transport [47]. Knockdown of ATG7 may limit erastin-induced ferroptosis with decreased intracellular ferrous iron levels, and lipid peroxidation [48]. In conclusion, most of these 14 genes in the signature are closely related to ferroptosis and play an important role in tumor development, which merits further study.

We further analyzed the underlying mechanisms of different prognosis between high- and low-risk groups. The GSEA results indicate that the enrichment pathways of the two groups are different. The high-risk group tends to be enriched in energy metabolism such as ribosomes, oxidative phosphorylation, and arachidonic acid metabolism. High-risk patients are likely to require the joint participation of the above pathways due to the inhibition of ferroptosis. Analysis of both groups of immune microenvironments found that, from the perspective of the ESTIMATE algorithm, the risk score was not correlated with the total number of immune cells and stromal cells. However, there are significant differences in the number of different types of immune cells in high- and low-risk groups from the perspective of the xCELL algorithm, and the high-risk group has significantly more CD8+ naive T cells and Th2 cells. In CRC, a substantial density of CD8+ T-cells in tumor tissue is generally associated with a favorable prognosis and sensitivity to chemoradiotherapy and immunotherapy [49, 50], partly because CD8+ T cells enhance ferroptosis-specific lipid peroxidation in tumor cells and thereby improve the anti-tumor effect of ferroptosis [51]. CD8+ naive T cells in the high-risk group were notably overexpressed, and further research may be required to explore the evolution process from naive to maturity. The TMB of the high- and low-risk groups was basically the same, which just showed that the ferroptosis risk score in this study was independent of the TMB level, and RC patients were grouped from a distinct perspective. Regarding the TIDE score, the dysfunction score was significantly greater in the high-risk group. The TIDE score is primarily used to measure tumor immune escape from tumor immune dysfunction. In clinical practice, a higher TIDE score was associated with poorer immune checkpoint blocking treatment and shorter survival [29]. This result shows that the immune dysfunction in the high-risk group is more serious, indirectly suggesting that there is a relationship between ferroptosis and the immune microenvironment, which needs to be further explored. Analysis of 34 immune checkpoints showed significantly lower expression of CD155 in the high-risk group, while no significant differences were found for the remaining checkpoints. CD155 gene is overexpressed, which begins at an early stage in tumorigenesis and continues to late stages in colorectal carcinoma [52]. Besides, CD155 interacts with TIGIT on natural killer cells and T cells may transmit inhibitory signals to immune cells [53]. The significantly lower expression of CD155 in the high-risk group suggests that this group of patients may have characteristics of insensitivity to immunotherapy.

Combined with the analysis of drug susceptibility in the database, it was suggested that patients in the high-risk group were more sensitive to certain drugs. Although the difference in IC50 was not too large, it still provided us with an idea that these drugs may inhibit RC by enhancing ferroptosis. AS605240 is a kind of phosphatidylinositol 3-kinase (PI3K) gamma inhibitor. Studies have shown that oral administration of AS605240 in a mouse colitis model can reduce intestinal inflammation in mice through a number of immunomodulatory effects [54]. MK-2206 is an inhibitor of Akt in the PI3K/Akt/mTOR pathway, and a multicenter phase II clinical study has demonstrated that its combination with neoadjuvant therapy can improve the pathological complete response (pCR) rate of HR-negative and HER2-positive breast cancer [55]. OSI-027 is an inhibitor of mTOR downstream of PI3K and can inhibit colon cancer cell growth through the _4_EBP1/eIF_4_E/PUMA pathway [56]. At present, studies have found that activation of the PI3K/Akt/mTOR signaling pathway can inhibit the ferroptosis of tumor cells through lipid production [57]. It has been reported that PI3K inhibitors can induce immunogenic ferroptosis in tumor cells, thereby achieving anticancer effects [32]. Therefore, PI3K pathway inhibitors induce ferroptosis by affecting the tumor immune microenvironment, and have broad research prospects in the field of colorectal cancer treatment. CI-1040 is a kind of MAPK inhibitor. An early multicenter phase II clinical study to explore oral CI-1040 in the treatment of multiple cancer types showed unsatisfactory results [58], so this medicine is basically eliminated. MP470 is a kind of receptor tyrosine kinase inhibitor, now known as amuvatinib [59]. It was used in a phase II clinical study of platinum-refractory small cell lung cancer patients, and the efficacy was not satisfactory [60]. QS11 is a kind of small molecule synergist with Wnt-3a ligand in the activation of Wnt/beta-catenin signal transduction [61]. A recent study on gastric cancer found that activation of the Wnt/beta-catenin signaling attenuates cellular lipid ROS production and subsequently inhibits ferroptosis [62]. Therefore, from the perspective of ferroptosis, QS11 may have the opposite effect. Studies have shown that the combined treatment of β-elemene and cetuximab is sensitive to KRAS mutant CRC cells by inducing ferroptosis, which will hopefully provide a prospective strategy for CRC patients with RAS mutations [63].

To further improve the clinical predictive value of the risk score, we combined common clinical prognostic factors (age and TNM stage) to construct a final prognostic model, which is composed of the risk score and age. 1-year, 3-year and 5-year OS were predicted, and the prediction accuracy was further improved compared with the risk score alone. We look forward to further discussion and promotion of the detailed mechanism of this model. In recent years, many clinical prognostic models have been built, involving various cancers and mechanisms [21, 64–66]. While the model presented in this study is still in its theoretical exploration stage, we hope that through this limited research it will provide a reference for more in-depth research on the mechanism of ferroptosis in the RC field in the future. There are also some obvious limitations in this study. Our cluster and DEGs analysis and subsequent risk model construction are two relatively independent parts. The former attempts to present different ferroptosis patterns in RC but is not discussed in detail. The latter is the main body of the article, which may confuse readers about the structure of this article. The data set selected for the bioinformatic analysis is itself a very small subset of RC patients. This subset inherently suffers from limited extrapolation, although we obtained acceptable results with the validation set. The sample size included in qRT-PCR is limited. If the sample size increases, the expression level of its mRNA may change on this basis.

## Conclusions

This study provides a risk score of FRGs for RC by analyzing the TCGA READ cohort from the perspective of FRDs, which can effectively distinguish the prognosis of two groups of patients and predict 1-year, 3-year and 5-year OS. The 14 genes involved in this risk score may affect the occurrence and development of RC. It may also affect drug sensitivity from ferroptosis, immune infiltration and other aspects, providing a reference for further in-depth discussion. We constructed a more complete prognostic model combined with the clinical characteristics of the patients, which enhanced the predictive ability of the risk score.

## Supplementary Information

**Additional file 1:** **Fig. S1.** (A) Consensus matrix legend of the cluster. (B-I) The heatmap of consensus matrix with k from 2 to 9. (J) Consensus cumulative distribution function (CDF). (K) Relative change in area under CDF curve. (L) Tracking plot of the cluster.

## Data Availability

The datasets generated and analysed during the current study are available in the TCGA repository, https://tcga-data.nci.nih.gov/tcga. The independent validation cohort data is available in the GEO repository, https://www.ncbi.nlm.nih.gov/geo (GSE87211).

## Abbreviations

CRC: Colorectal Cancer
RC: Rectal Cancer
TME: Total Mesorectal Exicision
nCRT: neoadjuvant Chemoradiotherapy
ctDNA: Circulating Tumor DNA
ICI: Immune Checkpoint Inhibitor
FRG: Ferroptosis-Related Gene
TCGA: The Cancer Genome Atlas
READ: Rectum Adenocarcinoma
GEO: Gene Expression Omnibus
HPA: Human Protein Atlas
IHC: Immunohistochemistry
DEG: Differentially Expressed Gene
t-SNE: t-distributed Stochastic Neighbor Embedding
FRDEG: Ferroptosis-related Differentially Expressed Gene
GO: Gene Ontology
KEGG: Kyoto Encyclopedia of Genes and Genomes
BP: Biological Process
CC: Cellular Composition
MF: Molecular Function
LASSO: Least Absolute Shrinkage and Selection Operator
K-M: Kaplan-Meier
ROC: Receiver Operating Characteristic
qRT-PCR: Quantitative Real-Time Polymerase Chain Reaction
GSEA: Gene Set Enrichment Analysis
MSigDB: Molecular Signatures Database
TMB: Tumor Mutation Burden
TIDE: Tumour Immune Dysfunction and Exclusion
OS: Overall Survival
BH: Benjaminiand Hochberg
GPX4: Glutathione Peroxidase 4
HR: Hazard Ratio
AUC: Area Under Curve
Fe-S: Iron-sulfur
MRP1: Multidrug Resistance-associated Protein 1
ABC: ATP-binding Cassette
PI3K: Phosphatidylinositol 3-Kinase
pCR: pathological Complete Response
