# Supplementary material for: Construction and validation of a novel Ferroptosis-related gene signature predictive model in rectal Cancer
Source: BMC Genomics. 2022 Nov 22;23:764. doi: 10.1186/s12864-022-08996-6 (PMC9682793; doi:10.1186/s12864-022-08996-6)
Supplement: Supplementary file 1 — Additional file 1: Fig. S1. (A) Consensus matrix legend of the cluster. (B-I) The heatmap of consensus matrix with k from 2 to 9. (J) Consensus cumulative distribution function (CDF). (K) Relative change in area under CDF curve. (L) Tracking plot of the cluster. [file 12864_2022_8996_MOESM1_ESM.docx]

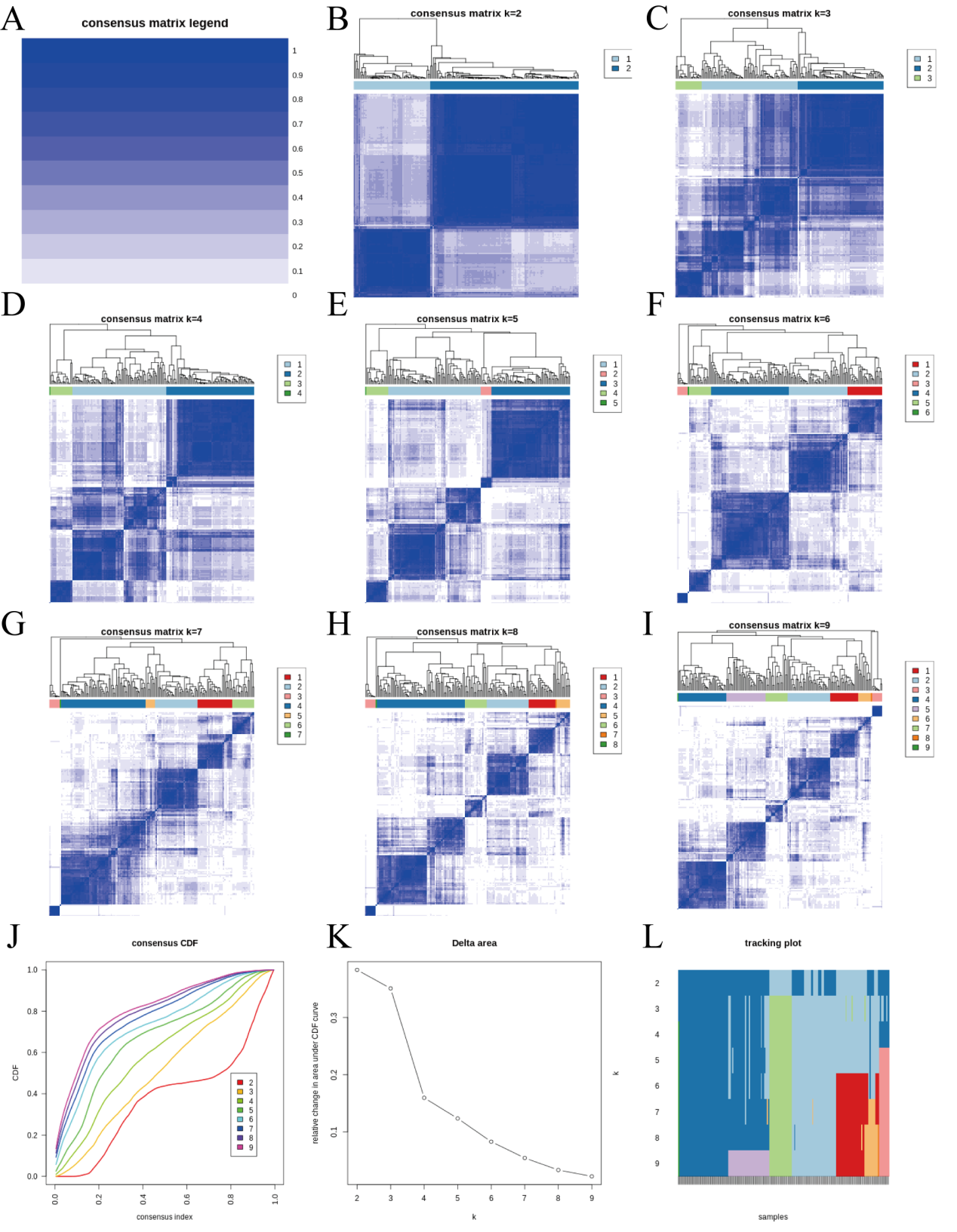


Supplementary Fig. 1. (A) Consensus matrix legend of the cluster. (B-I) The heatmap of consensus matrix with k from 2 to 9. (J) Consensus cumulative distribution function (CDF). (K) Relative change in area under CDF curve. (L) Tracking plot of the cluster.
